# Recent Advances in Liposome-Based Molecular Robots

**DOI:** 10.3390/mi11090788

**Published:** 2020-08-20

**Authors:** Kan Shoji, Ryuji Kawano

**Affiliations:** 1 Department of Mechanical Engineering, Nagaoka University of Technology, Kamitomioka 1603-1, Nagaoka, Niigata 940-2188, Japan; 2 Department of Biotechnology and Life Science, Tokyo University of Agriculture and Technology, Naka-cho 2-24-16, Koganei, Tokyo 184-8588, Japan

**Keywords:** molecular robot, giant unilamellar vesicles, synthetic ion channel, nanopore sensing, molecular motor, DNA computing

## Abstract

A molecular robot is a microorganism-imitating micro robot that is designed from the molecular level and constructed by bottom-up approaches. As with conventional robots, molecular robots consist of three essential robotics elements: control of intelligent systems, sensors, and actuators, all integrated into a single micro compartment. Due to recent developments in microfluidic technologies, DNA nanotechnologies, synthetic biology, and molecular engineering, these individual parts have been developed, with the final picture beginning to come together. In this review, we describe recent developments of these sensors, actuators, and intelligence systems that can be applied to liposome-based molecular robots. First, we explain liposome generation for the compartments of molecular robots. Next, we discuss the emergence of robotics functions by using and functionalizing liposomal membranes. Then, we discuss actuators and intelligence via the encapsulation of chemicals into liposomes. Finally, the future vision and the challenges of molecular robots are described.

## 1. Introduction

An artificial cell is a cell-imitating artificial system that exhibits characteristics of living cells, including evolution, self-reproduction, metabolization, and communication [1,2,3]. The development of artificial cells is one of the main objectives in the field of synthetic biology, and numerous studies have been reported. For example, gene expression [4], metabolic networks [5,6], growth and division [7,8], adaptivity [9], communications [10,11], and motility [12] of artificial cells have been reported. Although the recent developments of artificial cells successfully provided the potential for practical applications [13,14], the functioning of artificial cells has not yet reached a practical level.

Hence, another concept called “molecular robotics” has been proposed, which aims to offer practical uses in the fields of medicine, drug discovery, environmental science, food science, and energy science [15,16,17]. In this concept, the essential robotics components that are intelligence systems [18], sensors [19], and actuators [20,21,22,23] are developed by integrating various technologies, such as DNA nanotechnologies, synthetic biology, polymer chemistry, and robotics, and these components are implemented into a microcompartment using bottom-up approaches. Although these elements have been individually developed, with performances that potentially offer practical uses, the integration of these elements into a single system is still challenging. To integrate these three elements into a single robotics system, a compartment that can encapsulate these components is required. Giant unilamellar vesicles (GUVs) [24,25], DNA capsules [26,27], gels [28,29], and polymersomes [30] have been proposed as structures that may act as such compartments. The compartment of a molecular robot must be able to undergo membrane functionalization with membrane proteins and be able to facilitate the encapsulation of molecules in order to implement intelligence systems, sensors, and actuators. GUVs can, in particular, satisfy these requirements.

Here, we review recent advances in GUV-based molecular robot technologies, including methods of GUV production, the emergence of sensors by the functionalization of liposomal membranes, and the emergence of actuation and intelligence by encapsulating macromolecules into GUVs. In addition, we summarize the future outlook towards the integration of individual elements into a single compartment and the manufacture of a liposome-based molecular robot.

## 2. Giant Unilamellar Vesicles (GUVs) Generation Using Microfluidic Devices

Unilamellar vesicles (UVs) consist of a single lipid bilayer that forms a spherical shell shape in aqueous solution. UVs of 1–100 μm in diameter are called giant UVs (GUVs) and are traditionally prepared as a simplified membrane model of a cell. For application as molecular robots, GUVs must have a high stability, monodispersity of the droplet size, encapsulation efficiency, and productivity. Conventional methods for GUV formation include the gentle hydration method [31] and electro-formation method [32]. However, the GUVs formed by these methods cannot be applied to the development of molecular robots due to the lack of monodispersity in size and encapsulation efficiency. Recently, to overcome these issues, numerous microfluidic techniques to form GUVs have been reported.

**Figure 1:**
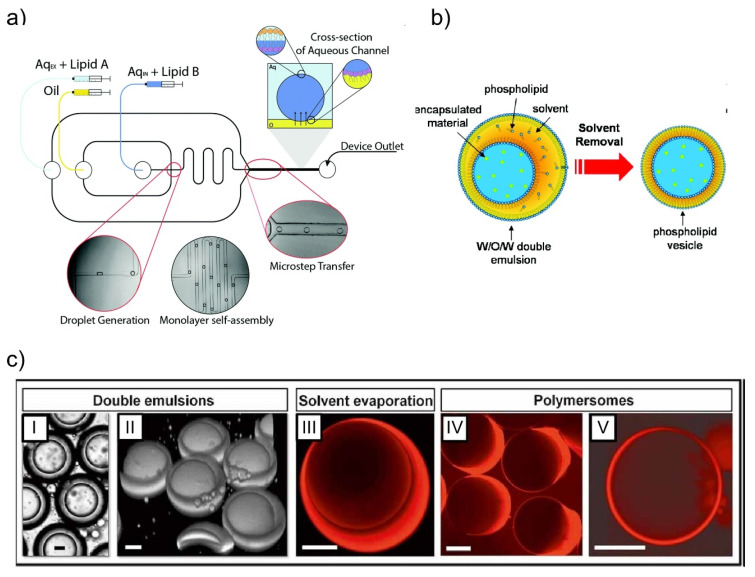
Schematic illustrations of giant unilamellar vesicles (GUV) and polymersome formation strategies. (**a**) The droplet transfer method. GUVs are formed by transferring w/o emulsions through the lipid monolayer. Reproduced with permission from [38]. (**b**) w/o/w emulsion template method. w/o/w emulsions are initially prepared by microfluidic channels. Then, GUVs are prepared by dewetting the oil solvent. Reproduced with permission from [41] (**c**) The w/o/w emulsion method can also be applied to the formation of polymersomes. (I) Bright-field microscope and (II) confocal microscopy images of w/o/w emulsions. (III) The organic solvent is labeled with Nile Red. After evaporating of the organic solvent, aggregates of excess polymer either (IV) remain attached to the polymersomes, or (V) occasionally detach from the polymersomes. Reproduced with permission from [52].

One such strategy involves a droplet transfer method via a microchannel [33,34]. In this method, water-in-oil emulsions (w/o emulsions) with phospholipids are first prepared, with GUVs being subsequently produced by the transfer of the emulsions through a lipid monolayer formed at an oil-water interface. To improve the yield and the monodispersity of the GUV size, microfabrication and microfluidic technologies have been applied. Because the uniformity of the GUV size is in relation to that of the w/o emulsion size, optimal w/o emulsions are formed by microfluidic methods instead of pipetting or vortexing. The Malmstadt group applied a T-junction microchannel to form w/o emulsions [35]. The w/o emulsions were formed by flowing an aqueous solution and oil into the T-junction microchannel. Then, the prepared droplet suspension was poured into the layered solution of the oil/lipid mixture and the aqueous solution. Finally, GUVs were generated by the droplet passing through the lipid monolayer at the oil-water interface. Because the experimental conditions for generating the w/o emulsions—including the capillary number, flow rates, and channel dimensions—allow the control of the droplet size, the monodispersity of the GUV size can be achieved. In addition, microfluidic channels for the on-chip generation of GUVs have also been reported [36,37,38]. In these microfluidic channels, the formation of w/o emulsions and GUVs by transferring w/o emulsions through the lipid monolayer are performed on the single microfluidic chip. First, w/o emulsions were formed by a cross-junction microchannel. Then, the w/o emulsions were transferred to the lipid monolayer prepared in the microchannels, facilitating the generation of GUVs (Figure 1a). Another approach to improve the monodispersity and productivity of GUVs is a microcapillary-based centrifugal microchip [39,40]. In this approach, a water-filled glass capillary was inserted into the oil layer of the oil-water layered microchamber. The droplets were effused from the capillary and transferred to the lipid monolayer by a centrifugal force. As a result, GUVs with a higher throughput and uniformity in size were formed when compared with other droplet-transferred methods, including microfluidic methods.

Another strategy to prepare GUVs using microfluidic technologies is by w/o/w emulsion template methods [41,42]. In this strategy, w/o/w emulsions with lipid monolayers are initially prepared using microfluidic channels, and the oil solvent is removed from the hydrophobic layer by the dewetting phenomenon. As a result, a lipid bilayer forms and generates GUVs (Figure 1b). The Weitz group first realized this strategy by using a mixture of toluene and chloroform as the organic solvent and evaporating it. The phospholipid concentration in the hydrophobic layer increases when the organic solvents are evaporated, with a depletion force being generated due to excess phospholipid molecules in the solvent. This induces the dewetting phenomenon. In this strategy, the method of dewetting significantly impacts the productive efficiency of the GUVs. The Dekker group reported the use of 1-octanol as the oil phase in the w/o/w emulsion to give a quick and clean physical solvent-extraction process [43]. On the other hand, the Huck group controlled the interfacial energies of w/o/w emulsion systems precisely by adding surfactant in the outer water phase to provide a successful control of the GUV formation processes, even in complex w/o/w systems including multicompartment emulsions [44].

Finally, a pulsed-jet flow method has been reported by the Takeuchi group [45,46]. In this method, GUVs are generated by applying a pulsed-jet flow to planar lipid bilayers analogous to the formation of soap bubbles from a soap film. Because the planar lipid bilayer formed by the droplet contact method (DCM) has a high stability [47,48], GUV generation is achievable without the rupture of the lipid bilayers. DCM is a droplet-based lipid bilayer formation method, where the lipid bilayers are formed by bringing into contact the lipid monolayers surrounding the droplets. Although in other strategies w/o emulsions are prepared before forming the GUVs, GUVs can be directly generated from the lipid bilayers in this strategy. As a result, the encapsulation efficiency is higher than in other strategies where the formation of w/o emulsions by vortexing, pipetting, or using microfluidic channels impacts on the encapsulation rate. In addition, by pre-forming asymmetric planar lipid bilayers and applying a pulsed-jet flow, asymmetric GUVs can be generated [46].

Moreover, to efficiently encapsulate and reconstitute proteins into the GUVs and the liposomal membrane, a microfluidic pico-injection technology that is based on droplet pairing with electro-coalescence has been applied to the bottom-up assembly of GUV-based artificial cells [49,50].

As stated above, numerous studies have reported on the improvement of the size-monodispersity, encapsulation efficiency, and yield of GUVs in the past decade, and the related technologies are maturing. In recent studies, DNA capsules [26,27] and polymersomes [30] were also proposed as potential compartments of molecular robots. Although GUVs have conventionally been used as the compartments of artificial cells, for molecular robots other materials may be considered that provide comparable or superior functions to GUVs. In fact, polymersomes have a higher durability than GUVs [51]. In addition, polymersomes can be efficiently prepared by a w/o/w emulsion template method similar to that of GUVs (Figure 1c) [52,53]. Furthermore, pore-forming proteins can be reconstituted into polymeric membranes [54,55], with this system already in practical use as a nanopore sequencer (MinION, Oxford Nanopore Technologies Ltd.). Because of these advantages of polymersomes, they have recently begun to be used as the compartments of artificial cell systems [56]. In the near future, it is likely that further research aimed towards the application of polymersomes and other capsule technologies to molecular robots will lead to their increased use as compartments.

## 3. Functions of Liposomal Membranes

One of the advantages of applying GUVs as the compartments of molecular robots is the possibility to add functions to the membrane by integrating proteins, peptides, and DNA structures. In addition, the semi-permeability of the lipid membrane can be exploited to prepare the concentrated encapsulation of chemicals or migration of GUVs. In this section, we explain the functions of lipid membranes applied in molecular robotics technologies.

### 3.1. Functionalization of Lipid Membranes

The main application of functionalized lipid membranes for molecular robots is as sensors, sensing being one of the elemental robot technologies. By integrating the lipid membrane with membrane proteins, peptides, synthesized ion channels, and DNA nano-structures, the membrane is able to sense target molecules or external conditions including pH, light intensity, pressure, viscosity, and temperature. Here, we explain functionalized lipid membranes and their application as sensors.

#### 3.1.1. Chemical Sensing

Ion channels, pore-forming proteins, and receptor proteins that are reconstituted into a cell membrane play special roles in the transportation of ions and molecules and as the sensors of specific molecules. Similarly for molecular robots, if these functions are embedded in the liposomal membrane at the interface between the robot interior and the exterior environment, they could act as sensors to monitor external conditions. Synthetic ion channels and nanopore technologies have a potential application in molecular robots for chemical detection because of their ability to transport ions and molecules [20].

**Figure 2:**
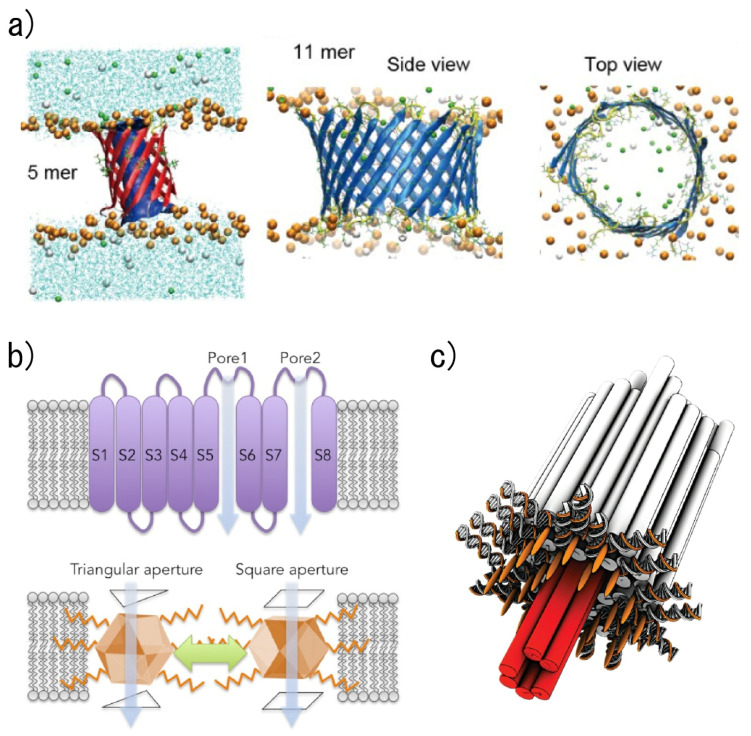
Schematic illustrations of synthetic ion channels. (**a**) A β-hairpin-based synthetic ion channel that forms a β-barrel structure in a lipid bilayer. Cyan lines indicate water molecules, and the lipid molecules were omitted for clarity (excluding phosphorus atoms as orange spheres). Green and white spheres indicate the potassium and chloride ions, respectively. Reproduced with permission from [60]. (**b**) A metal-organic cuboctahedra-based synthetic ion channel that presents two current states. Reproduced with permission from [64]. (**c**) A DNA origami nanopore that can be inserted into a lipid bilayer. Red denotes a transmembrane stem; orange strands with orange ellipsoids indicate cholesterol-modified oligonucleotides. Reproduced with permission from [65].

Synthetic ion channels are designed channel structures that can be inserted into the lipid bilayer and that can control ion transportation through the channel depending on the specific molecules and voltage, as well as other stimulations [57]. Hence, by analyzing changes in ion transportation, synthetic ion channels function as chemical sensors. Although numerous studies about synthetic ion channels have been performed since the 1980s by imitating and modifying motifs in nature, in this manuscript we focus on recent studies about *de novo*-designed synthetic channels, including the use of peptides, organic compounds, metal–organic scaffolds, and DNA. Because synthetic ion channels were initially designed by imitating pore-forming peptides, peptide-based synthetic ion channels have traditionally been studied by numerous groups. Recently, the DeGrado group reported on a *de novo*-designed ion-selective four-helical bundle peptide that transports Zn^2+^ and Co^2+^ ions, but not Ca^2+^, across lipid membranes [58]. The Baker group also recently reported *de novo*-designed protein ion channels that offer a stable structure by designing a hydrogen bond network with membrane-exposed residues [59]. Furthermore, we recently reported a *de novo*-designed β-hairpin peptide that can form large nanopore structures ranging from 1.6 to 6.2 nm, with the nanopore applied as a nanopore sensor that can detect double-stranded DNA (Figure 2a) [60].

The use of organic compounds also became one of the main ways to prepare synthetic ion channels when π-stacks were established as a fundamentally new structural motif for building synthetic ion channels. The Davis group demonstrated the transportation of Na^+^ ions by stacking G-quartets that were supported by π-stacking [61], while the Gong group successfully built an ion channel from a covalent, shape-persistent m-oligophenylethynyl (OPE) macrocycle [62]. Metal-organic channels were introduced by the Kim group in 2008 [63]. Such ion channels were formed from Metal-Organic Polyhedra-18 (MOP-18) that can transport protons and alkali-metal ions across lipid membranes. Furukawa and our group also recently reported metal-organic cuboctahedra-based synthetic ion channels that demonstrate two distinct conductance states, by embedding a single metal-organic porous molecule with the geometry of an Archimedean cuboctahedron into an artificially reconstructed lipid bilayer membrane (Figure 2b) [64].

Another strategy to develop synthetic ion channels utilizes DNA origami for the formation of nanopore structures. The Simmel group first reported a synthetic ion channel constructed by DNA origami techniques that could be inserted and fixed into the lipid bilayer (Figure 2c) [65]. In addition, DNA nanopores of various pore sizes [66,67,68] that can provide sensing functions (including for temperature [69], chemicals [70], and potential [71]) have recently been reported through the design and chemical modification of DNA sequences.

As mentioned above, various types of synthetic ion channels have been proposed, offering a potential as sensors of liposome-based molecular robots. In particular, DNA nanopores would offer advantages in sensing because several components of molecular robots are driven via the use of DNA or RNA signals as the trigger, and DNA nanopores are able to sense such DNA signals. Furthermore, in addition to DNA nanopores, various lipid bilayer-assisted DNA origami nanostructures [72,73,74,75] were also reported, and the functionalization of GUVs by DNA nanostructures would be expected. In the future, evaluations and improvements in the stability and durability of these channels will be required for their successful application in molecular robots as sensors.

Nanopore technologies also offer a potential in designing chemical sensors for molecular robots [76,77,78,79,80]. In this technology, pore-forming transmembrane proteins are reconstituted into the lipid bilayer, and a target molecule passes through the nanopore. In particular, nanopore sensors with pore-forming proteins such as α-hemolysin (αHL) [81] and Mycobacterium smegmatis porin A (MspA) [82] are commonly applied for the detection and analysis of single-stranded DNAs or RNAs. On the other hand, various DNA nanotechnologies, including DNA computing [83] and DNA origami [84], have been applied when manufacturing molecular robots. Therefore, nanopore technologies have attracted much attention in sensing for molecular robots.

**Figure 3:**
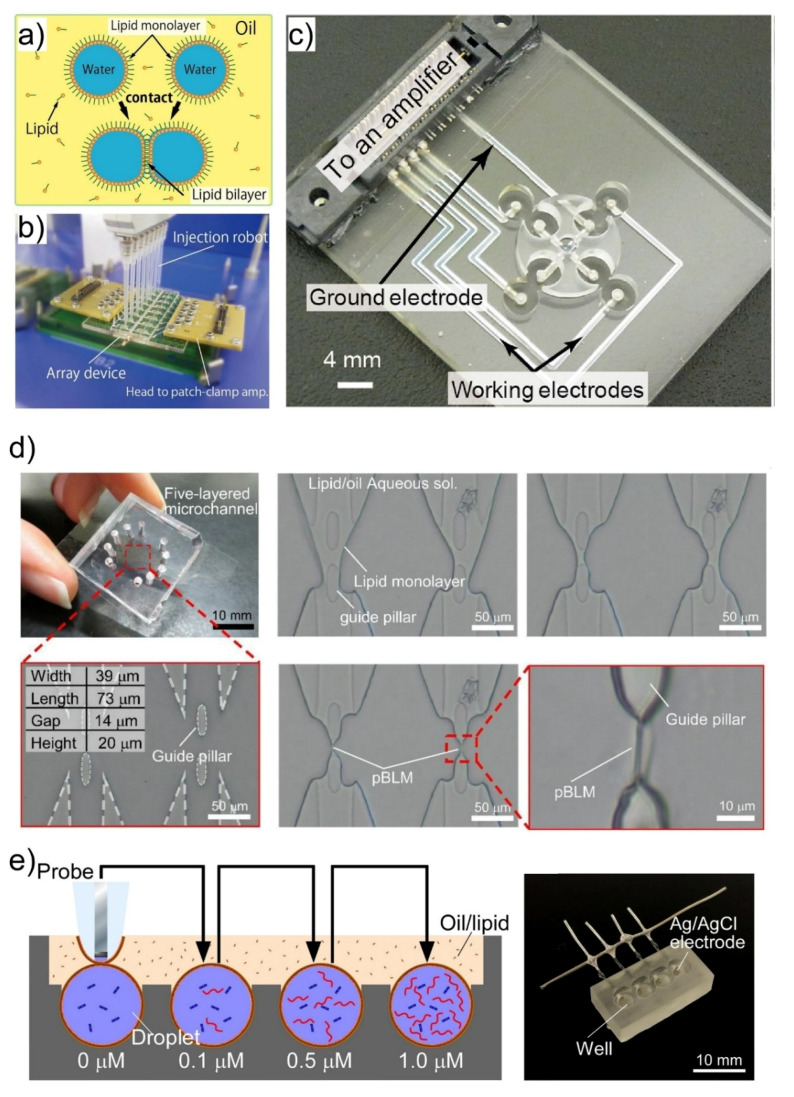
(**a**) A schematic illustration of the droplet contact method. (**b**) A multiple current recording device in which infinity-shaped chambers are arrayed. (**a**,**b**) are reproduced with permission from [48]. (**c**) A high-throughput channel current recording device in which lipid bilayers are formed and de-formed by rotating the chamber. Reproduced with permission from [87]. (**d**) A microfluidic-based multiple lipid bilayer formation. Lipid bilayers are formed by controlling the water-oil interface using the five-layered microfluidic channel. Reproduced with permission from [90]. (**e**) Lipid bilayer formation and channel current recording using an Ag/AgCl microelectrode. The lipid bilayer formation can be controlled by manipulating the microelectrode. Droplets including 0, 0.1, 0.5, and 1.0 μM ssDNA were prepared, and the channel currents were continuously measured by manipulating the probe. Reproduced with permission from [92].

Nanopore sensing technologies are applied to evaluate nanopore-based chemical sensors. In this method, chemical information can be obtained by measuring the change in the current when target molecules translocate through the nanopores reconstituted in a planar bilayer lipid membrane (pBLM). Although the painting method [85] and the Montal–Mueller method [86] have been conventionally used to prepare pBLMs, the stability and yield have been limited. In our group, we proposed nanopore sensing platforms based on a droplet contact method (DCM) (Figure 3a) to conveniently and efficiently perform pBLM formation and channel current measurements [48,87,88,89,90,91,92]. For example, the stability of the pBLM formed between two droplets was dramatically improved by utilizing a separator in which micro-scale holes were fabricated in a parylene film [48]. The fabrication of multiple chambers (Figure 3b) [48], a rotating system (Figure 3c) [87], and a microfluidic-based system (Figure 3d) [89] improved the throughput of the channel current recording. In recent developments, we developed micro- and nano-electrode-based nanopore sensing platforms in which pBLMs can be formed by simply pushing the electrode down into the layered bath solution of an oil/lipid mixture and an aqueous buffer solution (Figure 3e) [91,92].

**Figure 4:**
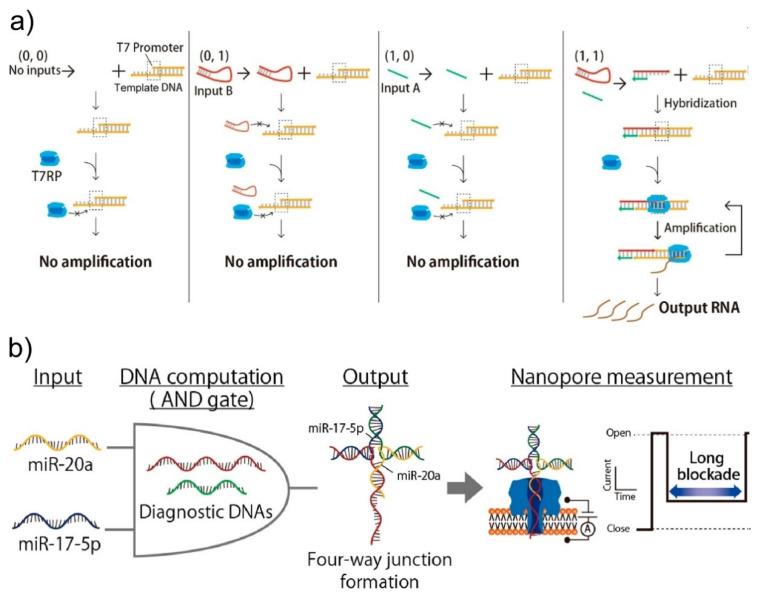
AND gates using DNA computing and nanopore technologies. The AND operation is detected as an increase of (**a**) the event frequency (reproduced with permission from [93]) and (**b**) the dwell time (reproduced with permission from [94]).

Because of our nanopore sensing platform having a higher membrane stability and throughput, applications of DNA nanotechnologies to nanopore sensing were achieved. Namely, we successfully created a logic gate [93] and detected specific microRNA [94,95] by combining DNA computing techniques. In this logic gate, the AND logic operation was provided by amplification and transcription from DNA to RNA using T7 RNA polymerase (T7RP). The calculation results were then detected by nanopore sensing as the event frequency of RNA translocations (Figure 4a) [93]. For cases in which two input DNAs exist in the droplet with the template DNA and T7RP, defined as input (1 1), two input DNAs form a duplex and the duplex hybridizes with the template DNA. Then, T7RP binds to the promoter region and synthesizes a large amount of RNA as output 1. In the other cases (inputs (0 0), (0 1), and (1 0)), the input DNA cannot hybridize to the template DNA, resulting in output 0. Furthermore, we also reported a simple detection method of multiple microRNAs (miRNAs) based on DNA/miRNA hybridization and nanopore sensing (Figure 4b) [95]. In this method, two template DNAs were designed to hybridize to miRNA-20a and miRNA-17-5p and construct a four-way junction. When the four-way junction was formed by these DNAs and miRNAs, the four-way junction could not translocate through αHL nanopores and became stuck in the pores, resulting in a long blocking current signal (Figure 4b). On the other hand, when both or one of the miRNAs were not present, the four-way junction was not formed, and the dwell time of DNA or miRNA in the nanopore became much shorter than in the case of the four-way junctions. Therefore, by analyzing the dwell times, this system can also work as an AND gate. As shown above, a combination of nanopore technologies and DNA computing could therefore facilitate the sensory function of molecular robots.

#### 3.1.2. External Condition Sensing

**Figure 5:**
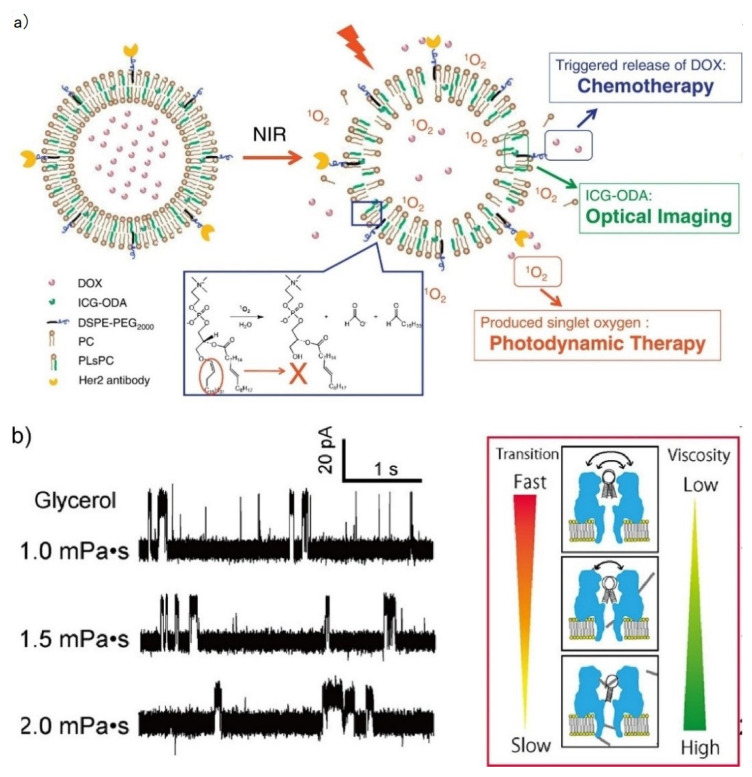
(**a**) Schematic illustration of a photo-sensitive liposome. Near-infrared light mediated specific drug release and synchronous photodynamic therapy and chemotherapy. Reproduced with permission from [101]. (**b**) A nanopore-based viscosity sensor. Transitions of current states are dependent on the viscosity. Reproduced with permission from [103].

To develop a drug release system in a GUV-based drug delivery, external condition-sensitive GUVs, including to pH, light, and thermal gradients, have conventionally been developed by modifying the liposomal membranes [96,97,98,99,100]. For example, liposomes formed by palmitoyl homocysteine, which can release encapsulated drugs in areas with a pH lower than the physiological pH, were reported by the Shinitzky group in 1980 [96]. Recently, not only could GUV rupture with a subsequent release of drugs be induced by pH-sensitive GUVs that consisted of oleic acid:DOPE, but membrane fusion could as well [100]. In addition, the photo-sensitive liposomes offer a combination of photodynamic therapy and chemotherapy by releasing drugs and producing reactive oxygen species (Figure 5a) [101]. The Schwille group recently reported pH-stimulated reversible shape changes in trapped GUVs, without compromising their free-standing membranes [102].

In another method, we reported nanopore sensing-based viscosity sensing [103]. In this sensing system, the viscosity can be analyzed by measuring the channel currents of the hairpin DNA-integrated αHL nanopore. In this sensor, the channel current shows three distinctive current signals (upper level, intermediate level, and lower level) that reflect the three different orientations of hpDNA in the vestibule [104], and the phase transitions between these three states are continuously observed. By analyzing the frequency of the fluctuation, the viscosity can be estimated (Figure 5b).

### 3.2. Use of Semi-Permeability of Lipid Membranes

**Figure 6:**
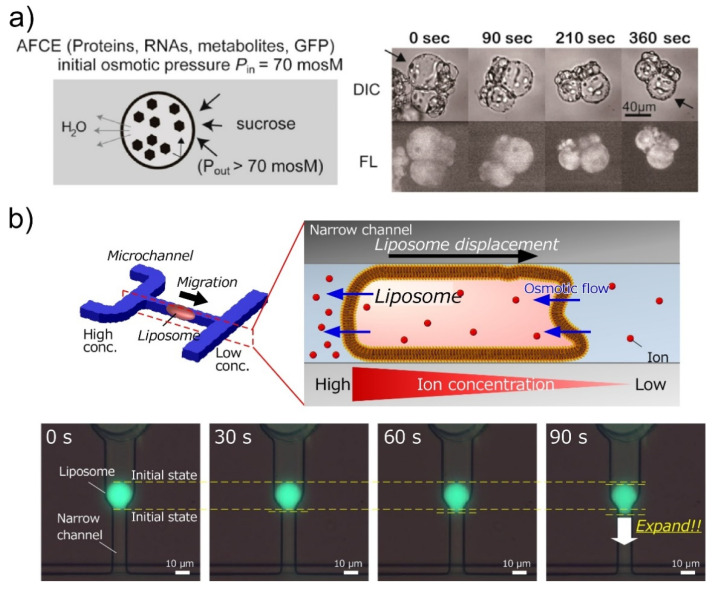
Applications of osmotic pressure to liposome-based molecular robots. (**a**) Control of the concentration of macromolecules in GUVs by an additive-free cell extract (AFCE). Reproduced with permission from [105]. (**b**) Osmotic-engine-driven GUVs in microchannels. Reproduced with permission from [107].

Another advantage of the lipid membranes in molecular robots is their semi-permeability. To manufacture cell-imitating molecular robots, it must be possible to encapsulate various molecules into the GUVs at a high concentration. However, GUVs with a concentrated inner solution could not be prepared by conventional GUV formation methods, as shown above. Hence, Fujiwara and Yanagisawa have proposed a method to concentrate the inner solution using hypertonic conditions (Figure 6a) [105]. In this method, the GUVs prepared by the droplet-transfer method were immersed in the outer solution with a higher osmotic pressure than the inner solution. As a result, the concentrations of macromolecules in the GUVs were controlled by the ratio of the osmotic pressure between the inner and outer solutions. Furthermore, Anges–Koback and Keating have reported a complete budding and asymmetric fusion of GUVs to produce daughter vesicles [106]. GUVs encapsulating a polyethylene glycol/dextran were prepared, and budding was induced by increasing the osmotic pressure.

In addition, the semi-permeability of GUVs was exploited in the migration of GUVs. We reported a GUV movement induced by an osmotic pressure difference (Figure 6b) [107]. We imitated a cell migration mechanism induced by osmotic pressure difference, which is called the “osmotic engine model” [108], and applied this mechanism to GUVs. In this model, a spatial gradient of ion channels forms an ion concentration difference, which results in a net inflow of water at the leading edge and a net outflow of water at the trailing edge. The GUVs were trapped tightly inside the microchannel, and the solution with different salt concentrations flowed to the front and rear of the GUV. As a result, the water passed through the lipid membrane from a lower to a higher salt concentration, and the GUV moved in the direction of the lower salt concentration, owing to the osmotic pressure difference.

## 4. Encapsulation in Giant Unilamellar Vesicles

Encapsulation is also a specific characteristic of GUVs. Because the lipid membrane allows the permeation of water and non-polarized small molecules, but not macromolecules or polarized molecules, various molecules can be encapsulated into the GUVs by adding them to the inner solution during GUV generation. In molecular robots, the encapsulated materials in the GUVs may be either drugs or materials that facilitate the functions of movement and intelligence.

### 4.1. Actuators of Molecular Robots

**Figure 7:**
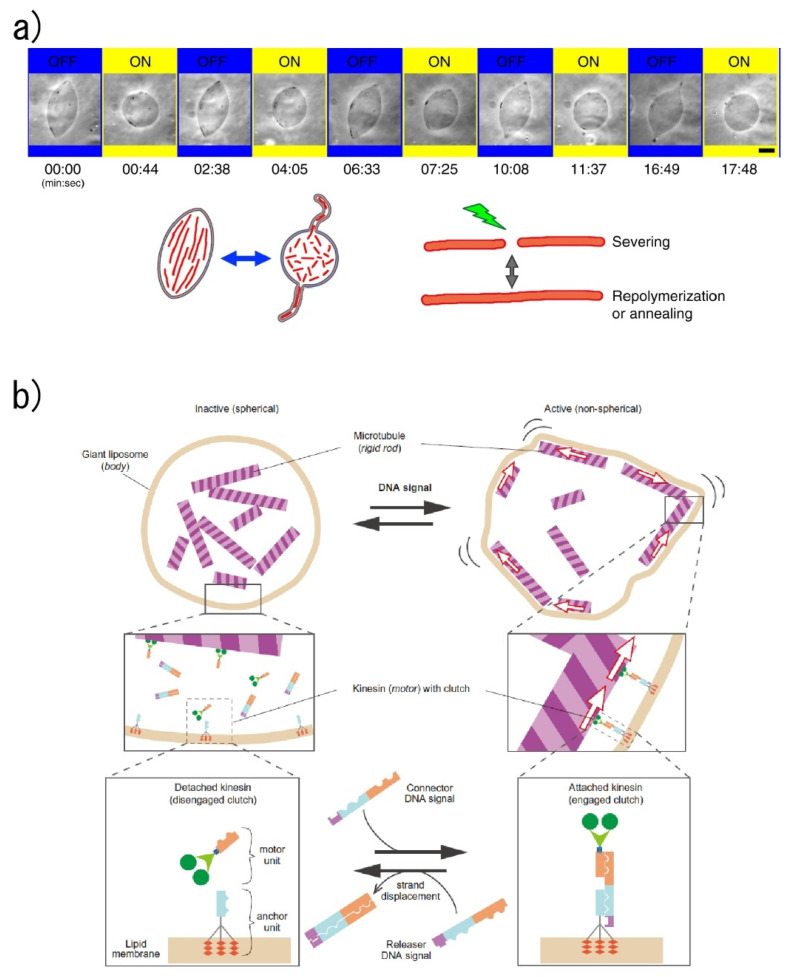
Emergence of GUV actuation by encapsulated molecules. (**a**) Reversible deformations of GUVs between spindle and sphere shapes by light irradiation of fluorescent-labeled actin. Reproduced with permission from [112]. (**b**) Controllable and programmable deformation of GUVs. Reproduced with permission from [113].

One of the main functions of the encapsulated materials is actuation. To achieve the concept according to which molecular robots act as microorganism-imitating microrobots, the encapsulation of molecular motors such as kinesin and myosin was performed. The Hotani group reported morphological changes of G-actin encapsulated GUVs by increasing the temperature, with the shapes of GUVs being dependent on the type of actin-crosslinking proteins due to the organization of their specific actin networks [109,110]. The Hayashi group also reported the transformation of GUVs induced by the encapsulated actin (Figure 7a) [111,112]. They successfully deformed the GUV by increasing the concentration of the encapsulated actin to a level comparable to that of the cytoplasm of living cells. In addition, reversible deformations of GUVs between spindle and sphere shapes were obtained by adjusting the osmotic pressure or by light irradiation of fluorescent-labeled actin. In a recent development, a controllable and programmable amoeba-like molecular robot was reported by the Sato group (Figure 7b) [113]. In this robot, microtubes, DNA-modified kinesins, and double-stranded DNAs are encapsulated into the GUVs, and linker DNAs with three cholesterol-modified DNAs are inserted into the inner liposomal membrane as anchors. When connector DNAs exist in the GUVs, the kinesins and the anchors are engaged. As a result, the microtubes can glide via the membrane-connected kinesins, and the shape of the membrane can be continuously changed.

Although it is still challenging to achieve a motility of molecular robots analogous to living cells—such as migration and chemotaxis—because it is difficult to complexly align the proteins in the GUVs like living cells, the switching techniques of activation and in-activation offer a potential control of actuation in molecular robots. As the next step, the conversion of the force generated by molecular motors into the movement of GUVs must be researched, and an efficient mechanism for this must be developed. Regarding other strategies, self-propelled techniques using bubbles, the Marangoni effect, interfacial tension, and nanofiber growth have also been reported [114,115]. To improve the mobility of molecular robots, we propose that a combination of encapsulated molecular motors and other technologies, such as self-propelled mechanisms, would be most efficient.

### 4.2. Intelligence of Molecular Robots

In robots, intelligence plays a significant role as the interface between sensors and actuators. Once sensors installed in the membrane sense external signals, robots must determine a response. In the field of molecular robotics, DNA computing technologies [83,116] and DNA-based calculation systems, including isothermal cascade reactions of DNAs [117], are proposed as intelligence systems.

**Figure 8:**
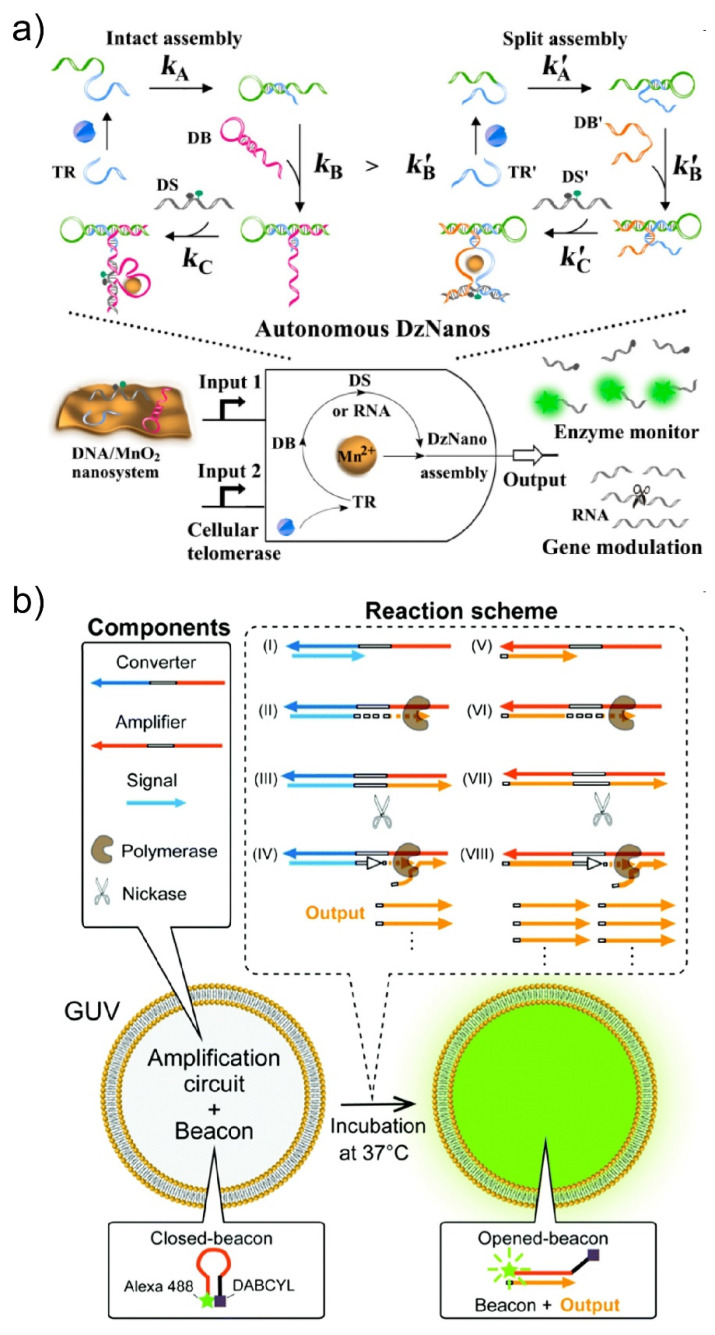
(**a**) A DNA-based calculation nanodevice that can act in living cells. Reproduced with permission from [108]. (**b**) An isothermal amplification circuit for specific DNA molecules that is implemented in GUVs. Reproduced with permission from [110].

DNA computing is an area of natural computing first reported by Adleman in 1994 [83], based on the principle that polynucleotides contain information encoded in the amino acid sequence, with this information being capable of being transferred and/or copied using chemical and enzymatic reactions. DNA computing was initially proposed as a parallel processing method that could calculate faster than conventional silicon-based computers. However, because of issues with low accuracy and a time-consuming data output process, the application of DNA computing is shifting towards computation that can work in biological conditions. In fact, DNA computing-based calculation methods in living cells have been reported (Figure 8a) [118,119]. To perform DNA computing and obtain the calculated result in molecular robots, the isothermal amplification of DNAs is needed. The Komiya group has reported an isothermal cascade reaction that can exponentially amplify pre-designed single-stranded DNA, ultimately aiming for the development of DNA-based computing systems that can drive molecular robots [120]. Furthermore, the Sato group successfully performed the isothermal amplification of specific single-stranded DNAs in GUVs (Figure 8b) [121].

The emergence of intelligence in molecular robots still faces challenges because intelligence does not emerge until it connects sensors and actuators and works as a controller. Clarification is required regarding what signals will work for the input and output of molecular robots and for the subsequent design of appropriate intelligence systems.

## 5. Conclusions and Future Outlook

In this review, we focused on recent advances in liposome-based molecular robots. We described microfluidic methods of GUV generation and the emergence of sensors, actuators, and intelligence systems via the functionalization of liposomal membranes and the encapsulation of chemicals into GUVs. To date, the efficient generation of GUVs with a size-monodispersity and chemical encapsulation ability has been achieved by using microfluidic techniques. In addition, DNA capsules and polymersomes offer a potential as the compartments of molecular robots. Regarding the sensors, synthetic ion channels and nanopore sensing technologies are useful for sensing external chemicals. In particular, recent developments of *de novo*-designed peptide, protein, and DNA channels can provide stable structures with good size controllability, and these may attract attention for use as sensors of molecular robots. Nanopore sensing has recently offered specific chemical detection by combining DNA computing techniques, and this system has also provided both sensory and intelligence functions. Furthermore, viscosity measurements have been achieved by using hairpin DNA-integrated αHL nanopores. Regarding the actuators, encapsulated molecular motors can facilitate the deformation of GUVs, with the actuation being controlled by specific stimulations. In the future, we will look towards a mechanism in which the actuation of molecular motors will be efficiently converted into migratory motion. It is proposed that the intelligent control of molecular robots can be achieved with DNA computing technologies and the isothermal amplification of DNA. Because intelligence plays a role as the interface between sensors and actuators, an intelligence system should be designed and optimized for applications in molecular robots.

Finally, molecular robots are microorganism-imitating robots aimed towards a practical usage in the fields of medicine, drug discovery, environmental science, food science, and energy science in a manner distinct from artificial cells. However, it is still challenging to integrate all the robotics systems into a single compartment because it is necessary to design and construct each of the functions, including sensors, actuators, and intelligence, so that they do not interfere with each other and can act in a single GUV. In the present state, molecular robots have not shown a clear distinction from artificial cells. As a field, we should aim to develop robotic systems that can provide superior functions and abilities to cells by combining various technologies that encompass not only synthetic biology but also mechanical, electrical, chemical, and information engineering. We believe that the establishment of molecular robotics methods would offer a game-changing technology in these various fields.
